# Audit of the change in the on-call practices in neuroradiology and factors affecting it

**DOI:** 10.1186/1471-2342-6-13

**Published:** 2006-10-16

**Authors:** Nitin Mukerji, Dorothy Wallace, Dipayan Mitra

**Affiliations:** 1 Department of Neurosurgery, Newcastle General Hospital, Newcastle-upon-Tyne, NE4 6BE, UK; 2 Department of Neuroradiology, Newcastle General Hospital, Newcastle-upon-Tyne, NE4 6BE, UK

## Abstract

**Background:**

On call practices had recently changed at the Newcastle General Hospital to accommodate increasing CT scan requests and reduce the workloads of the radiologists. In the new system, the person responsible for dealing with the out of hours requests for imaging changed from the neuroradiologist to the neuroradiographer. This audit was conducted to assess any change in the departmental workload as a result of this change.

**Methods:**

The audit was carried out over a period of six months and data was collected from the on-call booklets which the neuroradiographers maintained and the log books maintained in the department of neuroradiology. Details of the imaging requested; the source of the request, the reason for the request and the results of the scans were recorded and analysed using Microsoft Excel™.

**Results:**

The number of CT scans requested from the A&E went up by 73.4% after the change in practice and majority of these increases were due to increased requests for scans on head injuries which increased by 122%. Although this was not statistically significant due to lack of study power, it is clinically relevant.

**Conclusion:**

The increase in the number of CT scans for head injuries reflects a general change in practice in management of head injuries in the UK. Changing the gatekeeper from radiologist to radiographer was associated with an increase in CT rate, particularly for head injuries. Other factors such as clinician seniority and a greater awareness of the NICE guidelines may have also contributed.

## Background

The department of neuroradiology at the Newcastle General Hospital runs a very busy practice, even out of hours. Primarily responsible for this are the facts that Newcastle General Hospital is the regional referral centre for Neurosurgery and also has one of the busiest Accident and Emergency (A&E) departments in the region on site. Given the already high workloads, the practice of assessing on-call requests for imaging was changed to reduce the workload of the neuroradiologist. Previously, out-of-hours (outside of 0800 to 1700 hours, Monday to Friday) imaging requests from the A&E and other non-neurosciences departments on-site were made to the on-call neuroradiologist who assessed the appropriateness of the same. From 15/1/2004 a provision was made for the neuroradiographers to accept these calls directly without going through the on-call neuroradiologist. In order that this system did not result in appropriate CT scans being performed, the on-call neuroradiographers were issued with booklets to keep a detailed log of all requests for purposes of review and audit. A protocol for the indications of out-of-hours imaging was also drawn up and distributed amongst the neuroradiographers for reference. The neuroradiologist on-call was contacted only if the neuroradiographer was not fully satisfied with the appropriateness of the request as per the protocols.

The department of neuroradiology works along side a department of general radiology at the Newcastle General Hospital and requests for neuroimaging i.e. CT head and neck scans, MRI scans and angiograms from all departments at the Newcastle General Hospital and all imaging requests like CT head scans, chest x-rays, cervical spine x-rays for inpatients and MRI requests from neurosciences are handled by the department. Separate radiographers are on-call for both general and neuroradiology. It is worth mentioning that the new practice of receiving calls by the neuroradiographer was the usual practice for requests from within the neurosciences departments (i.e. neurosurgery and neurology) and it was merely extended to all departments on-site.

This audit was aimed at assessing the change in the departmental workload as a result of this change in practice and also to collect more information about the general out of hours working pattern in the department.

## Methods

The study was carried out over a period of six months from 15/10/03 to 15/4/04 to include two three month periods, one before (15/10/03 to 14/1/04, period A) and one after the introduction of the new system (15/1/04 to 15/4/04, period B). Data was collected from the on-call booklets mentioned previously as well as from the log books maintained in CT and MRI suites. The latter source was used to collect all the data before the introduction of the booklets and also for any additional information after the introduction of the booklets. A total of 74 booklets were collected in the three month period between 15/1/04 to 14/4/04. 16 booklets were missing and all the data pertaining to these were collected from the CT and MRI suite log books. Details of the type imaging, the source of the request, the reason for the request and the results of the scans were recorded. All the data was recorded on to a Microsoft Excel™ worksheet for analysis. One sample t-test was used to compare the numbers before and after the change. The study was approved by the neurosciences departmental ethics committee and conformed to the Helsinki declaration. There were no competing or financial interests.

## Results

### On-call working pattern

**Figure 1:**
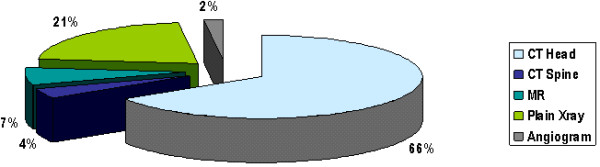
A diagrammatic representation of the break-up of the examinations performed between 15/1/04 to 15/4/04 (n = 507).

Data for the overall work pattern was collected from the on-call booklets. A total of 507 examinations were performed over 74 days during the study period in which the on-call booklets were introduced ie. 15/1/04 to 15/4/04. Average number of examinations out-of-hours were 6.8 per day with a range from 1 to 18. Out of all these examinations, 90 were performed between midnight and 7 am resulting in an average of 1.2 calls per night. CT head scans accounted for 66% of the total workload with plain x-rays accounting for 21%, MRI 7%, CT spine 4% and diagnostic angiography 2% (Figure 1). A total of 336 CT head scans were performed out of hours in this period (mean 4.5/day, range 1–11). A&E department accounted for the maximum, 62% of the total number of CT requests. The neurosciences departments accounted for 29% and the others for 9%.

### Change in the workload before and after the introduction of the new system

**Figure 2:**
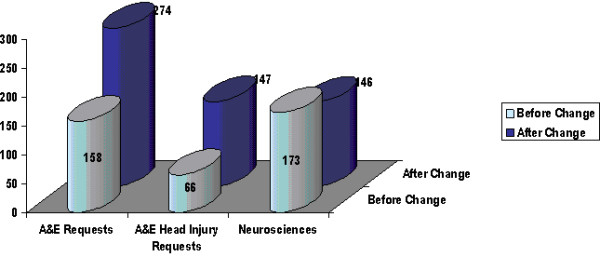
A diagrammatic representation of the change in referral patterns over the two three month periods included in the study.

This was assessed on the basis of CT head requests before and after the introduction of the new system. This data was collected from the CT scan suite logbook and had no missing data. The total number of CT scan requests from A&E had gone up from 158 before the change to 274 after the change, an increase of 73.4% (p = 0.17, 95% CI of difference of mean -520 to +952). This included all CT head requests from A&E, head injuries and otherwise. In the corresponding period the number of CT head requests from the neurosciences department went down from 173 before the change to 146 after the change, a decrease of 15.6% (p = 0.054, 95% CI of difference of mean -12 to +331). CT scans performed from head injuries from the A&E increased from 66 in the period between 15/10/03 to 14/1/04 to 147 between 15/1/04 and 15/4/04 an increase of 122% (p = 0.23, 95% CI of difference of mean -408 to +621) (Figure 2).

### Head injury scans

Of the 158 CT scans requested from the A&E between 15/10/2003 and 14/1/2004, 66 were requested for head injury (41.7% of all requests). The actual CT scan reports were traced and reviewed and there was one missing record. Findings related to an acute head injury were found in 15 patients (23%). Of these, surgically relevant findings like acute subdural haematomas, extradural haematomas, depressed skull fractures etc. were found in 8 patients (12.3%). Two patients had foreign bodies inside the skull, which might be considered surgically relevant.

In the period between 15/1/04 to 15/4/04, 274 CT head scans were requested from the A&E and 147 of these were for head injuries (53.8% of all requests). Reports of 138 out of the 147 head injury scans were traced and reviewed. Findings related to an acute head injury were found in 16(11%) patients. Surgically relevant findings were found in 7 patients, which represent 5% of the total number scanned for head injury.

## Discussion

This audit revealed important facts regarding the out-of-hours functioning of the department of neuroradiology. It is noteworthy that CT head scans performed for head injuries from the A&E went up in this period when the change in practice of handling calls for out-of-hours imaging was implemented and almost half of the scans requested were for head injuries. Although the trend of increase in A&E head injury CT scans with the advent of NICE guidelines is consistent with other published studies [1-5], the results fall short of statistical significance. This was probably because the lack of study power, which was one of the limitations of our study. Several factors could account for this increase in CT scan rates. A&E attendances during this period were 17163 with 618 head injuries during period A and 16339 with 726 head injuries during period B. Increased number of head injuries seen during period B could be one of the factors responsible for the rise in A&E CT scans. Contributory to the increase in the number of CT scans requested from A&E for head injuries may be the fact that junior medical staff at the A&E change during the first week of February and it is only expected that with new and probably less experienced medical staff the threshold for requesting a CT scan for head injuries would be low especially in the presence of established guidelines for scanning. How much this would be contributory to the increase in the number of scans can only be answered in a study which does not involve the changeover period which generally occurs twice a year in the beginning of February and August. A definitive way of establishing this would be to look at A&E scans in this period from previous years, however this data was not available as part of the audit as the objective of this audit was to examine the consequences of changing the investigation gatekeeper from radiologist to radiographer.

A greater awareness of the NICE head injury guidelines [1] for CT scans in head injuries also may have contributed to the rising numbers of head injury CT scans. These guidelines were officially released in June 2003 and A&E departments all over the UK have become increasingly aware of them and have tried to implement them. This has not been without its associated problems with radiology and out-of hours staffing and workloads. The main change with the NICE guidelines was that CT should replace skull x-rays as the screening tool in most patients with head injuries [2]. This has been received with mixed reaction over most parts of the UK with concerns over increasing numbers of CT scans and cost implications [3-8]. Investigators have tried to analyse retrospectively, existing head injury databases to determine which additional patients would have required CT scans according to the NICE guidelines [3,9]. There is a general consensus that the number of CT scans has increased two to fourfold as a result of the NICE guidelines but a cost benefit analysis taking into account the reduction in skull x-rays and number of admissions revealed an actual saving for the trusts [10].

The actual protocols used by the radiographers for accepting scan requests were based on the NICE guidelines, which were released in June 2003. The fact that a neuroradiographer working with a set of protocols would have less clinical knowledge and judgment than a neuroradiologist may have resulted in accepting more calls and performing more scans which if routed through a neuroradiologist may have been refused or waited till the next day. The radiographers worked with a set of guidelines which were based on the NICE guidelines and hence more accommodating towards head injury scans whereas the neuroradiologist prior to that worked on clinical experience and no specific guidelines. One must realize that this was the primary change in the working practice which was audited and the NICE guidelines may have affected the results indirectly.

## Conclusion

To conclude, this audit has revealed that the number of CT scans requested for head injuries is on the rise and with a general change in practice in the management of head injuries all over UK; this is likely to remain so and increase even further. Changing the gatekeeper from radiologist to radiographer was associated with an increase in CT rate, particularly for head injuries. Other factors such as clinician seniority and a greater awareness of the NICE guidelines may have also contributed. This has resource implications for most departments trying to cope with this change in clinical practice in managing head injuries. Regular audits need to be performed to make sure that resources are appropriately utilized.

## Abbreviations

A&E – Accident and Emergency, NICE – National Institute for Clinical Excellence, UK – United Kingdom, CT – Computed Tomography

## Competing interests

The author(s) declare that they have no competing interests.

## Authors' contributions

DM and DW conceived the study and carried out the data collection and analysis. NM prepared the manuscript, carried out the analysis and data interpretation. All authors read and approved the manuscript.

## Pre-publication history

The pre-publication history for this paper can be accessed here:
